# PD20 - Hospital admissions for food-induced anaphylaxis in Italian children: a new report for the years 2006-2011

**DOI:** 10.1186/2045-7022-4-S1-P20

**Published:** 2014-02-28

**Authors:** Rita Nocerino, Ludovica Leone, Vincenza Pezzella, Gianluca Terrin, Riccardo Troncone, Roberto Berni Canani

**Affiliations:** 1 Department of Translational Medicine-Pediatric Section, University of Naples “Federico II”, Naples, Italy; 2 Department of Women’s Health and Territorial Medicine, University "La Sapienza", Rome, Italy; 3 European Laboratory for the Investigation of Food Induced Disease (ELFID), University of Naples “Federico II”, Naples, Italy

## Background

During the last decade an increased incidence of food induced anaphylaxis has been demonstrated in Western Countries. We reported an increased incidence of hospital admission for food induced anaphylaxis in Italy from 2001 to 2005. We aimed to explore if this trend persists investigating the number of hospital admissions for food-induced anaphylaxis in Italy.

## Methods

The Italian Ministry of Health database was asked about hospital admissions for food-induced anaphylaxis from the year 2006; data were available up to year 2011. We identified hospital admissions for food-induced anaphylaxis using the following specific codes: ICD-9 codes: 99560, 99561, 99562, 99563, 99564, 99565, 99566, 99567, 99568, 99569. We investigated the total number of ordinary and day hospital admissions over the 6 years period, and the type of food responsible for the disease. The number of deaths for food-induced anaphylaxis was also recorded.

## Results

A total of 3121 admission for food-induced anaphylaxis (1454 ordinary admissions and 1667 day hospital; 57.7% male; mean age 14.28 years; minimum 0-maximum 92 years; 2253 in Northern Regions and 868 in Southern Regions) occurred during the 5 years study period. For the age 0-14 years, a total of 2252 admissions for food-induced anaphylaxis (1044 ordinary admissions e 1208 day hospital) occurred during the 5 year study period. In the same age group, a continuous increasing trend was observed: in the year 2006 the total number of hospital admissions for food-induced anaphylaxis was 270, comparing to 479 admissions for the year 2011 (+77.4%). The foods responsible for anaphylaxis in this age group were: cow’s milk (36.3%), hen’s egg (21.1%), nuts and seeds (3%), fruits and vegetables (2.9%), fish (2.3%), peanuts (1.7%), crustaceans (1.1%), other specified foods in 7.1%. anaphylaxis caused by food additives was reported in 0.4% of subjects, and for 24% of cases the responsible food was unavailable. We identified 4 deaths for food-induced anaphylaxis all occurred at hospital and in patients aged >14 years. The food responsible were peanuts, crustaceans, fruits and vegetables; in one case the food responsible was not identified.

## Conclusions

A persistent increasing pattern for food-induced anaphylaxis occurred in the last decade in Italy. Our data suggest the importance of more research to investigate the causative factors and the necessity to improve the healthcare service for this condition.

